# A new in vitro mouse oligodendrocyte precursor cell migration assay reveals a role for integrin-linked kinase in cell motility

**DOI:** 10.1186/s12868-016-0242-2

**Published:** 2016-02-01

**Authors:** Ryan W. O’Meara, Sarah E. Cummings, John-Paul Michalski, Rashmi Kothary

**Affiliations:** Ottawa Hospital Research Institute, 501 Smyth Road, Ottawa, ON K1H 8L6 Canada; Department of Cellular and Molecular Medicine, University of Ottawa, Ottawa, ON K1H 8M5 Canada; Department of Medicine, University of Ottawa, Ottawa, ON K1H 8M5 Canada; University of Ottawa Centre for Neuromuscular Disease, Ottawa, ON K1H 8M5 Canada

**Keywords:** Mouse oligodendrocyte precursor cells, Migration, Integrin-linked kinase, Extracellular matrix, Laminin, Fibronectin, Polylysine

## Abstract

**Background:**

The decline of remyelination in chronic multiple sclerosis (MS) is in part attributed to inadequate oligodendrocyte precursor cell (OPC) migration, a process governed by the extracellular matrix (ECM). Elucidating the mechanisms underlying OPC migration is therefore an important step towards developing new therapeutic strategies to promote myelin repair. Many seminal OPC culture methods were established using rat-sourced cells, and these often need modification for use with mouse OPCs due to their sensitive nature. It is of interest to develop mouse OPC assays to leverage the abundant transgenic lines. To this end, we developed a new OPC migration method specifically suited for use with mouse-derived cells.

**Results:**

To validate its utility, we combined the new OPC migration assay with a conditional knockout approach to investigate the role of integrin-linked kinase (ILK) in OPC migration. ILK is a focal adhesion protein that stabilizes cellular adhesions to the extracellular matrix (ECM) by mediating a linkage between matrix-bound integrin receptors and the cytoskeleton. We identified ILK as a regulator of OPC migration on three permissive substrates. ILK loss produced an early, albeit transient, deficit in OPC migration on laminin matrix, while migration on fibronectin and polylysine was heavily reliant on ILK expression.

**Conclusions:**

Inclusively, our work provides a new tool for studying mouse OPC migration and highlights the role of ILK in its regulation on ECM proteins relevant to MS.

## Background

Oligodendrocytes (OLs) are responsible for generating myelin, a lipid-rich structure that envelops central nervous system (CNS) axons, allowing for rapid communication between neurons. OLs generate myelin as they differentiate by extending multiple processes that contact adjacent axons, forming concentric wrappings of lipid-rich OL membrane mostly devoid of cytoplasm and stabilized by myelin structural proteins [1]. Prior to the onset of myelination, newborn OL precursor cells (OPCs) undergo a transient period of local proliferation followed by migration [2], their direction and extent governed by patterning molecules. Proper myelination is therefore highly dependent on the capacity of OPCs to migrate to target destinations and initiate the differentiation program. As such, researchers have endeavored to dissect the extracellular cues and the intracellular machinery governing OPC migration. This is especially relevant in neurological diseases like multiple sclerosis (MS) where the capacity for endogenous repair may be limited partly by the hampered migration of OPCs into lesions [3].

Over the years, our understanding of OL biology has vastly improved through the study of rat OLs in vitro, for which several isolation methods exist [4–6]. However, these strategies are often not ideal for isolating mouse OPCs for a number of reasons. First and foremost, mice possess fewer OPCs due to their smaller size. Primary mouse OPCs are also more difficult to isolate when compared to rat OPCs (reviewed by [7], and are less viable in vitro [8]). As such, researchers have endeavored to modify existing rat OPC protocols/assays to allow for their use with mouse-derived cells [7, 9–11] to take advantage of the broad spectrum of transgenic mouse lines.

We were interested in developing a mouse-optimized assay for studying OPC migration in vitro. Traditionally, OPC migration was typically assessed with the “transwell” or “agarose drop” assays [12]. The transwell assay [13] measures cell migration through a porous membrane, whereas the agarose drop assay [14] measures radial migration out of a drop of low-melting temperature agarose. While effective with rat cells, certain aspects of these assays limit their applicability to mouse OPCs. In particular, the agarose drop assay calls for relatively long assay durations (days), which does not appease the limited viability of mouse OPCs. Both protocols also call for large numbers of OPCs, which are laborious to obtain from mice, thereby fuelling us to devise a method for assessing mouse OPC migration that is rapid and requires few cells.

We previously established a protocol for isolating mouse OPCs [9], and as a byproduct of the procedure, OPCs tend to form aggregates (henceforth termed OPCAs) suspended in the culture media. We show that a highly enriched population of OPCs efficiently emerges from OPCAs, and describe a method to quantifiably assess this phenomenon. By combining the OPCA assay with conditional knockout genetics, we reveal a role for the focal adhesion protein integrin-linked kinase (ILK) in OPC migration on three substrates. Inclusively, we provide a new tool for the study of mouse OPC motility in vitro, and validate its utility through use in identifying ILK as a mediator of OPC migration.

## Methods

### Transgenic animals

Animals used for this work were cared for according to the Canadian Council on Animal Care guidelines under University of Ottawa Animal Care Committee protocol number OGH-130. The floxed *Ilk* mouse line (*Ilk*^*fl/fl*^; [15]) was graciously provided by Dr. René St-Arnaud (McGill University, Montreal, Canada). *Ilk*^*fl/fl*^ mice were subsequently bred to homozygosity with the *mT/mG* reporter mouse strain [16] to yield the *Ilk*^*fl/fl*^*; mT/mG* line.

### Cell culture

**Fig. 1:**
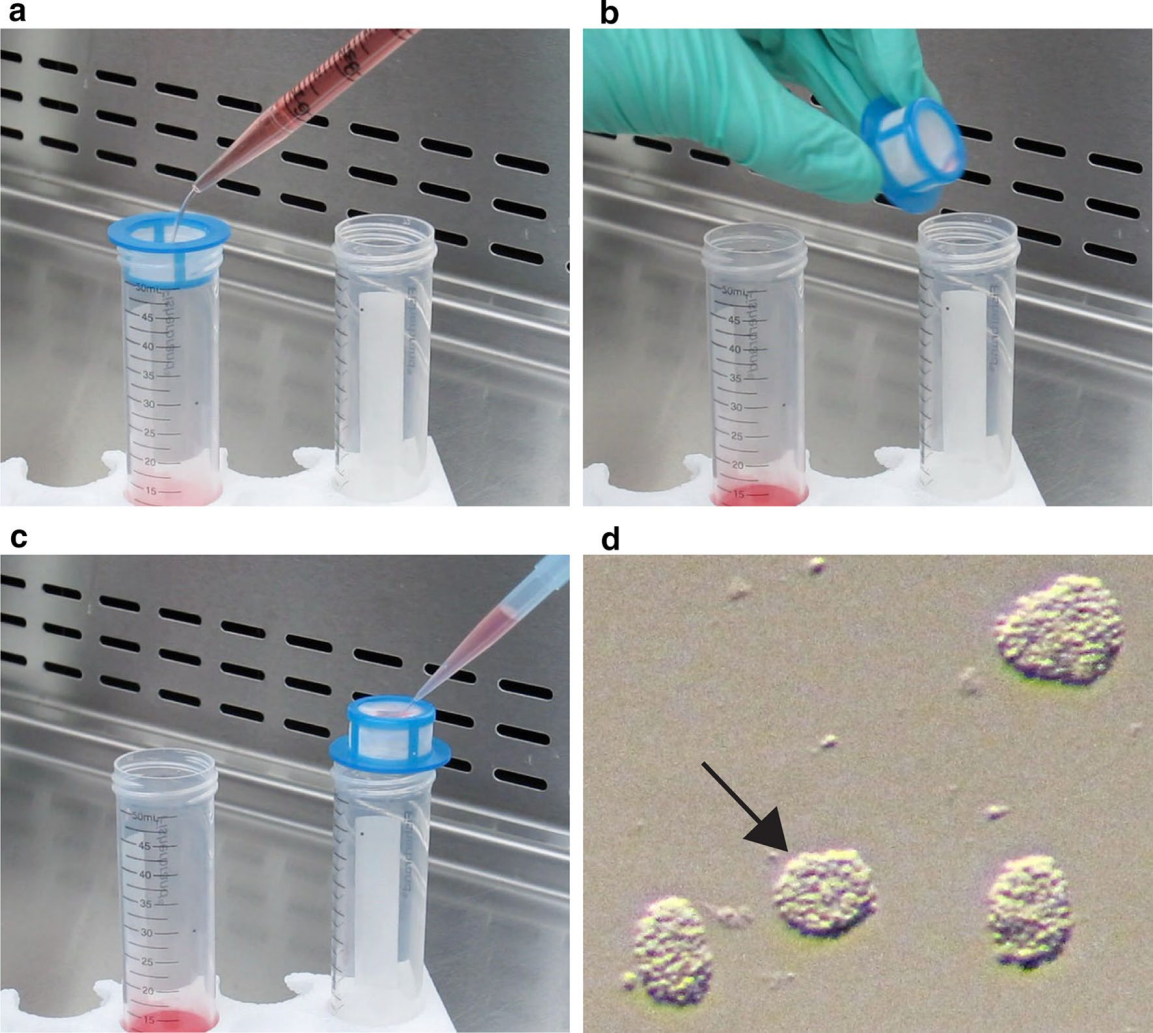
Pictorial demonstration on how to obtain OPCAs from cell suspensions. **a** The cell suspension collected from shaken mixed glial cultures is gently added to a 40 µm cell strainer over a 50 mL conical tube. **b** The strainer is then flipped upside-down and placed over a new 50 mL conical tube. **c** Migration media is used to backwash the strainer, causing the transfer of the OPCAs to the conical tube. **d** An image obtained through the eyepiece of a stereomicroscope, showing freshly isolated OPCAs. When selecting OPCAs for migration experiments, it is desirable to select those most uniform in dimension, such as the one denoted by the *black arrow*

OPCAs were generated as a byproduct of the mouse OPC purification protocol previously described by us [9]. Neonatal mouse cortices were dissociated and seeded into poly-l-lysine coated T25 flasks in mixed glial culture media. Once cultures had achieved appropriate density, the flasks first underwent rotary shaking utilizing a VWR Advanced 3500 Orbital Shaker (0.75 inch orbit) at low speed (50 rpm) for 1 h to remove contaminating microglia. Fresh mixed culture media was added back to the cultures, which were then shaken at a speed of 220 rpm overnight (approximately 16 h), to generate suspended clumps of OPCs (OPCAs). The media was passed through a 40 µm cell strainer and the OPCAs were subsequently backwashed onto sterile Petri dishes (Fig. 1a–c). OPCAs were picked from the Petri dish with a P20 pipette by aid of a stereomicroscope, and seeded individually onto substrate-coated coverslips in migration or differentiation media (see below). Coverslips were coated with either human placental merosin (laminin; 10 µg/mL; Millipore), fibronectin (50 µg/mL; Calbiochem) or >300,000 molecular weight poly-d-lysine (0.1 mg/mL; Sigma-Aldrich).

### Cell culture media

Mixed glial culture media was Dulbecco’s modified eagle medium (DMEM) supplemented with 1 % GlutaMAX (Life Technologies), 10 % fetal bovine serum (FBS), 0.33 % Penicillin–Streptomycin (P/S). From culture day 6 and onwards, the mixed glial culture media was supplemented with 5 µg/mL insulin.

Migration media was composed of 48–72 h conditioned mixed glial culture media (0.22 µm filtered), supplemented with 2 % B27 (Gibco), 100 µg/mL bovine serum albumin (BSA), 5 µg/mL insulin, 0.5 µg/mL Holo-transferrin, 60 ng/mL progesterone, 400 ng/mL 3,3′,5-triiodo-l-thyronine, 400 ng/mL l-thyroxine, 16 µg/mL putrescine, 5 ng/mL sodium selenite, 10 ng/µL platelet-derived growth factor (PDGF-AA; PeproTech), 50 ng/µL ciliary neurotrophic factor (CNTF; Peprotech) and 1 µg/mL aphidicolin (Sigma-Aldrich).

### Conditional genetic ablation

In vitro recombination of the *Ilk*^*fl/fl*^ and *mT/mG* alleles was achieved by treating mixed glial cultures with recombinant His-TAT (trans-activator of transcription)-NLS (nuclear localization sequence)-Cre recombinase (henceforth called TAT-Cre; Excellgen Incorporated). Briefly, the media was removed and the cultures were washed with PBS. TAT-Cre (or the corresponding vehicle control) was administered at 10 µL/mL in DMEM with 2 % B27 and incubated on the cultures for 1–2 h. This treatment was performed twice 48–72 h prior to OPCA isolation to promote a high level of recombination.

### Immunostaining and microscopy

OPCAs were fixed with 3 % paraformaldehyde (PFA) and then either processed for indirect immunofluorescence microscopy, or mounted onto slides in DAKO medium after staining with Hoechst.

For indirect immunofluorescence, fixed OPCAs were permeabilized with 0.1 % Triton X-100 for 10 min at room temperature (RT), and washed with PBS. OPCAs were blocked with 10 % goat serum, and incubated overnight at 4 °C with primary antibodies against Olig2 (EMD Millipore) or PDGFR-α (provided by Dr. William Stallcup, Sanford Burnham Medical Research Institute, La Jolla, California). The following day, the samples were washed with PBS and incubated with Alexa fluor-conjugated secondary antibodies (invitrogen) for 45 min to 1 h at RT. Coverslips were subsequently washed with PBS, counterstained with Hoechst and mounted onto slides in DAKO medium.

Standard epifluorescent images were acquired with an Axio Imager M1 microscope equipped with an AxioCamHR HRm Rev.2 camera using EX Plan-Neofluar 10x/0.3 Ph1 or Fluar 5x/0.25 M27 objectives and Axiovision 4.8.2 software. Confocal microscopy was conducted with a Zeiss LSM 510 Meta DuoScan microscope using EC Plan-Neofluar 10×/0.3 M27, 20×/0.5 M27, 40×/1.3 Oil DIC M27 or Plan-Apochromat 63×/1.4 Oil DIC M27 objectives and Zen 8.0 software.

Phase contrast microscopy was performed with an Axiovert 200 M microscope fitted with an AxioCamHR HRm Rev.2 camera using LD Plan Neofluar 20×/0.4 Korr Ph2 or EC Plan-Neofluar 10×/0.25 Ph1 objectives and Axiovision 4.6 software.

### Migration quantification

Phase contrast images of OPCAs were acquired at time zero to record their original diameter. Migration assays were fixed at 4, 10 or 24 h and imaged by fluorescence microscopy. Using Photoshop, “exclusion zones” were digitally overlaid onto the residual OPCA core, serving to define the starting point of migration; only cells beyond the exclusion zone were considered to have truly migrated. For OPCAs seeded on laminin and fibronectin substrates, the size of the exclusion zone was 1.5 × the OPCA diameter at time zero. For the poly-d-lysine substrate, the exclusion zone was 1.75×, 2.0× and 2.75× the time zero OPCA diameter for 4, 10 and 24 h time points, respectively.

Concentric rings were then digitally centered over the exclusion zone, their diameter increment being 50, 100 and 200 µm for 4, 10 and 24 h time points for assays conducted on laminin. For the fibronectin substrate, the ring diameter increment was 20, 40 and 80 µm for the 4, 10 and 24 h time points, respectively. For the poly-d-lysine substrate, the ring increment for the 4, 10 and 24 h time points was 10, 20 and 40 µm, respectively. The number of OPCs that migrated to each concentric ring was quantified and represented as the percentage of OPCs in each ring. This can be considered a measure of migration distance or be represented as the total number of migrated OPCs, regardless of distance. We interpret this latter parameter as a function of the cell to initiate migration or polarize.

### Immunoblot

Mixed glial cultures were cooled on ice for 3 min, rinsed with ice cold PBS, and scraped into a commercial RIPA lysis buffer (cell signaling) supplemented with 2 mM PMSF. Lysates were left on ice for approximately 2 min, and centrifuged at 15,000 rpm for 5 min. Clarified lysates were transferred to new microfuge tubes and stored at −80 °C.

For SDS-PAGE, 30 µg of cell lysate per sample was resolved on a poly-acrylamide gel, and transferred to a PDVF membrane at 0.25 amps for 70 min. Membranes were blocked for approximately 1 h at RT with 5 % skim milk power in TBST (10 mM Tris–HCl pH 8.0, 150 mM NaCl, 0.1 % Tween-20). Primary antibodies against ILK (cell signaling) and GAPDH (Abcam) were incubated overnight in blocking buffer. Membranes were washed with TBST and probed with HRP-conjugated secondary antibody for 45 min. Membranes were then washed with PBS, and treated with ECL (Thermo Scientific). Resulting films were scanned with an EPSON Perfection 2450 PHOTO scanner, imported into ImageJ, and a box of standard dimensions was placed over each band to measure the mean gray value. Densitometric values for ILK were normalized to GAPDH as a loading control.

### Statistical analysis

One “n” (i.e., experiment) was considered as data obtained from pooled biological material (i.e., cells) from mouse pups (usually 4–6) of a distinct litter. Differences in migration distance were assessed using two-way repeated measures ANOVA paired with Bonferroni multiple comparisons tests. All other data was tested using either unpaired two-tailed Student’s *t* tests or one-way ANOVA paired with Bonferroni multiple comparisons tests. For all statistical tests, “n” was equal to or greater than three, and differences in the mean were considered significant when p < 0.05.

## Results

### An assay for investigating mouse OPC migration in vitro

In 1980, McCarthy and DeVellis published a seminal paper describing the isolation and propagation of primary rat OPCs in mixed glial cultures. In this method, neonatal cortical tissue is dissociated into a single cell suspension and seeded into tissue culture flasks. Over several days, astrocytes form a monolayer on the base of the flask, upon which OPCs proliferate. The OPCs are relatively loosely attached to this monolayer, and are susceptible to separation when flasks are shaken on an orbital rotator overnight at 37 °C. This procedure renders the suspension of OPCs in the culture medium, many of which form aggregates (OPCAs) due to their innate tendency to do so [12].

In our mouse adaptation of the McCarthy and deVellis method [9], we took advantage of this aggregation phenomenon to assess OPC migration. After the overnight shaking step, the medium containing the OPCAs was filtered through a 40 μm cell strainer, and then backwashed with migration media into a 50 mL conical tube (Fig. 1a–c). The backwashed media was then transferred to a Petri dish, and OPCAs were picked with a micropipette by aid of a stereomicroscope. As a result of the mechanism of their formation, OPCAs can be heterogeneous in shape, and care was taken in selecting the most uniform aggregates for migration experiments (Fig. 1d).

Once selected, OPCAs were seeded individually onto substrate-coated coverslips in 48–72 h conditioned mixed glial culture media supplemented with various OL survival factors (see “Methods” for details). The purpose of this media was to favor the maintenance of OPCs in a precursor state, as migration generally ceases in differentiated OLs. We also aimed to control OPC proliferation by including aphidicolin (mitotic inhibitor) in the migration media, as excessive proliferation could give the false impression of extensive migration.

**Fig. 2:**
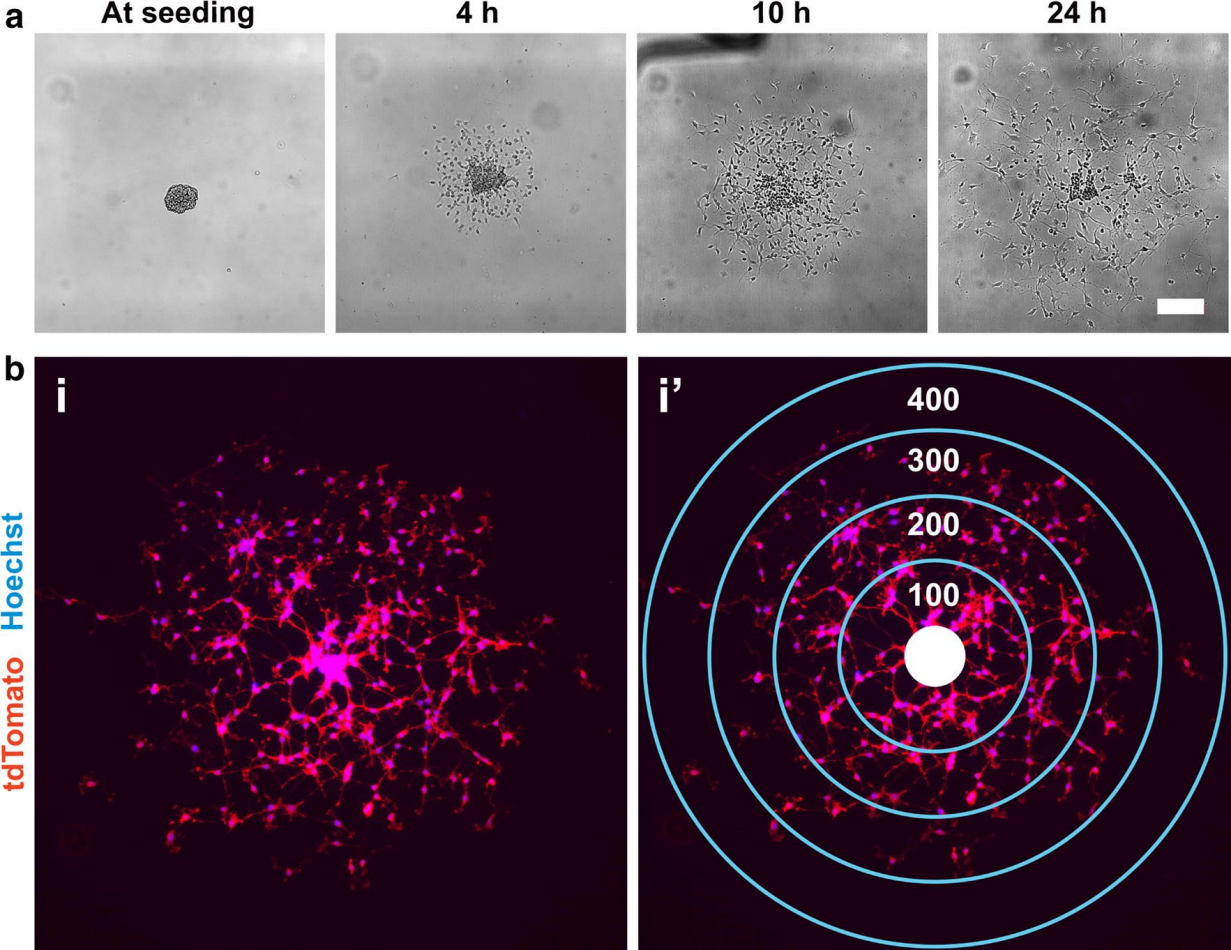
Seeding OPCAs on migration-permissive substrates yields a quantitative means to assess OPC motility. **a** Time-lapse phase contrast microscopy of an OPCA from which OPCs emerge radially over a 24 h period. **b** A 10 h migrated OPCA before (*i*) and after (*i’*) superimposing concentric rings (100 µm increments) over the residual OPCA core (*solid white circle*). Migration distance can be conveyed as the proportion of cells in each ring or the total number of migrated cells regardless of distance. Membrane-targeted tdTomato (*red*) facilitates visualization of cell bodies. *Scale bar*, **a** 50 µm

When seeded onto laminin-coated coverslips, large numbers of cells migrated out of OPCAs over a 24 h period (Fig. 2a). After a given experimental duration (e.g., 10 h), OPCAs were processed for immunofluorescence microscopy, and concentric rings (centered on the OPCA core) were digitally overlaid on the acquired images (Fig. 2b). When OPCs in each concentric ring were counted, distributions could be generated that described the net distance traveled by the cells over a given timeframe. Slow migration rate would cause OPCs to appear accumulated in rings proximal to the OPCA core, whereas efficient migration would increase the proportion of cells in the more distal rings. This data could also be portrayed as the total number of migrated OPCs (regardless of distance), which we interpret as a measure of migration initiation. For example, a reduction in the total number of migrating OPCs subsequent to an experimental manipulation could be interpreted as compromised migration initiation.

**Fig. 3:**
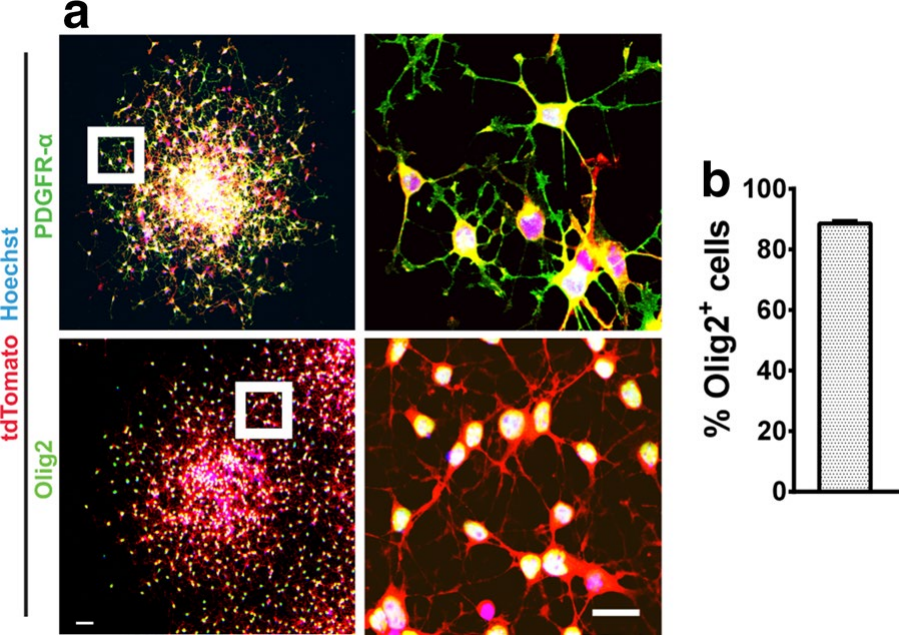
OPCAs are predominantly composed of OPCs. **a** 10 h migrated OPCAs showing the majority of migratory cells are positive for the OPC markers PDGFR-α and Olig2. **b** OPCAs are composed of on average 88.6 % (±0.4 %) Olig2^+^ cells. Data represent the mean ± SEM (n = 3). *Scale bars*, **a** 50 and 20 µm (*inset*)

To ensure that migratory cells were in fact OPCs, OPCAs were labeled with antibodies against the OPC markers platelet-derived growth factor receptor-α (PDGFR-α) and Olig2 (reviewed by [17]). Preliminary observations suggested the majority of cells were immunopositive for these markers (Fig. 3a), and subsequent quantification revealed that 88.6 % (±0.4 %) were in fact Olig2^+^ (Fig. 3b). Our observations indicate the majority of cells migrating from OPCAs are of OL-lineage, thereby validating the utility of this assay for studying OPC migration.

### Conditional ablation of ILK from OPCAs

**Fig. 4:**
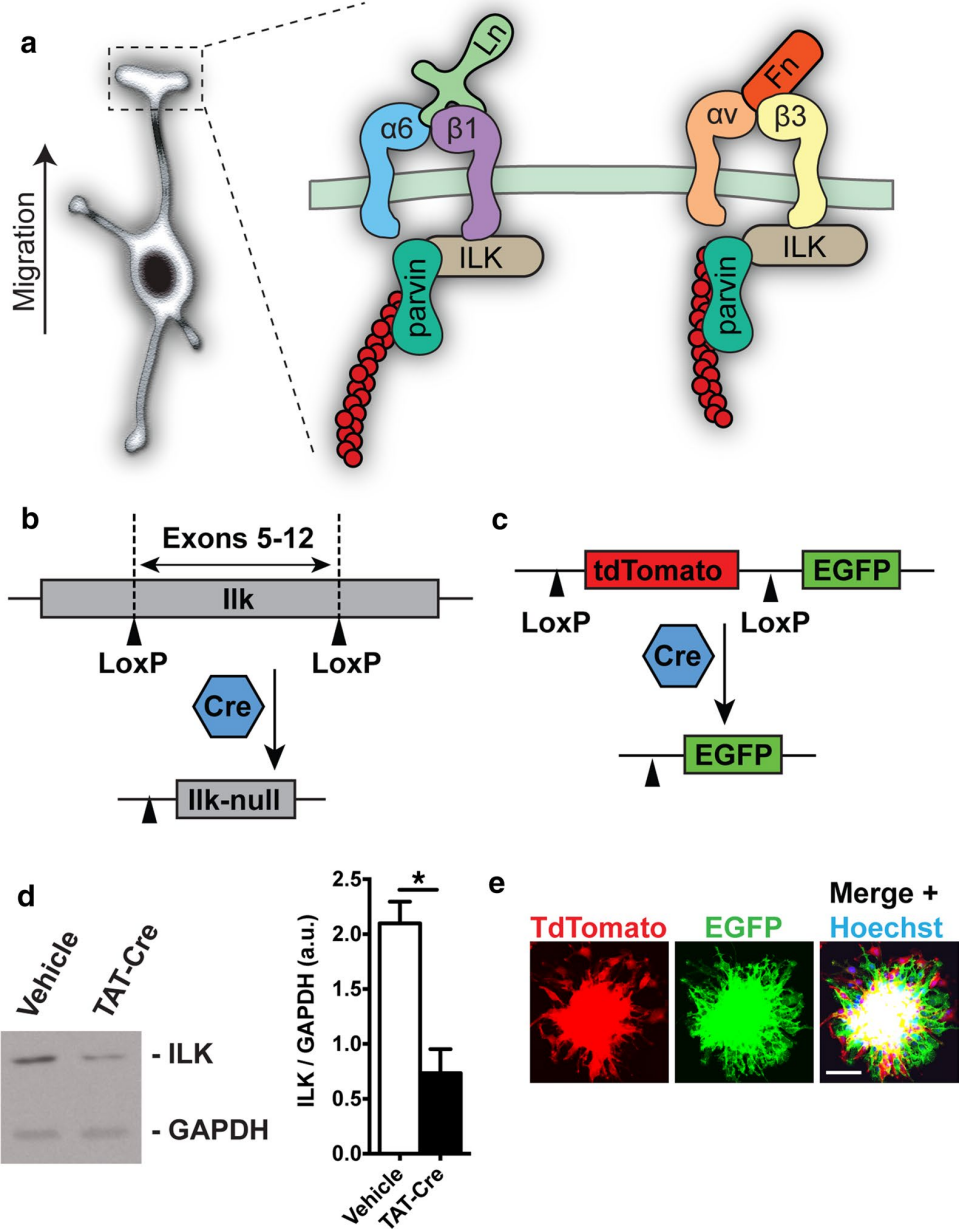
Conditional genetic ablation of *Ilk* from OPCAs. **a** ILK is a focal contact protein that links ECM-bound integrin receptors to the actin cytoskeleton via its partner parvin. In the depiction, α_6_β_1_ and α_v_β_3_ integrin receptors reside at the leading edge of a migrating OPC’s process, where they respectively bind laminin (Ln) and fibronectin (Fn). At the β-integrin cytosolic tails, ILK stabilizes ECM adhesions to the actin cytoskeleton (*red circles*) by acting as a scaffold. **b** Schematic representation of the mouse *Ilk* ^*fl/fl*^ locus, where Cre recombination excises exons 5–12 of the *Ilk* gene [15]. **c** Schematic representation of the *mT/mG* reporter locus [16]. Cre-recombination at the LoxP sites excises tdTomato and induces EGFP expression. **d** Western blot showing TAT-Cre reduces ILK protein when administered to *Ilk* ^*fl/fl*^ *;mT/mG* cultures. Significant difference confirmed by densitometric analysis. **e** OPCAs isolated from TAT-Cre treated *Ilk* ^*fl/fl*^ *;mT/mG* cultures, showing they are composed of both recombined (EGFP-positive) and non-recombined (tdTomato-positive) OPCs. Presumably, ILK is depleted from OPCs expressing EGFP. For **d**, data represent the mean ± SEM (n = 3), *p < 0.05 (Student’s *t* test). *Scale bar*, 50 μm

Subsequent to its validation, we aimed to utilize the OPCA assay to investigate the role of ILK in mouse OPC migration. ILK is a focal adhesion protein that elicits its function via interaction with the integrins, a family of transmembrane receptors that mediate adhesion to the ECM. During migration, integrin receptors cluster in discrete foci at the leading edge of lamellipodia, forming cell/matrix contacts that are used as anchor points for the actin cytoskeleton. Integrins are heterodimers composed of an α and a β subunit, and OL-lineage cells express α_v_β_1_, α_v_β_3_, α_v_β_5_, α_v_β_8_ and α_6_β_1_ integrins inclusively [18]. ILK binds to the intracellular tails of β_1_ and β_3_ integrin subunits [19, 20], and plays both signaling and scaffolding roles at actin-integrin connections (Fig. 4a; [21, 22]. ILK performs these functions with help from binding partners PINCH and parvin, which respectively link ILK to receptor tyrosine kinases and the actin cytoskeleton [23]. In OLs, ILK mediates cellular morphology by way of regulating actin architecture and the activity of the cytoskeletal remodeling protein RhoA [24, 25]. As cellular motility relies on Rho-GTPase signaling, cytoskeletal dynamics and morphological plasticity, we hypothesized that ILK is required for ECM-directed OPC migration.

To this end, we employed a mutant mouse possessing loxP sites flanking exons 5–12 of the endogenous *Ilk* gene (*Ilk*^*fl/fl*^; Fig. 4b), where recombination presumably produces null alleles [15]. We then bred *Ilk*^*fl/fl*^*;mT/mG* double transgenics by crossing *Ilk*^*fl/fl*^ mice with the *mT/mG* strain; a reporter line harboring a locus coding for near-ubiquitous expression of the fluorescent protein tdTomato [16]. The action of Cre recombinase at this locus ablates tdTomato, while at the same time inducing EGFP (Fig. 4c), thereby permitting identification of Cre-recombined cells. Therefore, recombined cells should express EGFP and be devoid of ILK, while non-recombined cells remain tdTomato^+^ and retain ILK expression.

Mixed glial cultures were then established from *Ilk*^*fl/fl*^*;mT/mG* mice and treated with cell-permeable TAT-Cre recombinase or vehicle control (see “Methods” for details). As expected, TAT-Cre administration led to a reduction in total ILK protein in *Ilk*^*fl/fl*^*;mT/mG* cultures as compared with vehicle-treated controls (Fig. 4d). We then isolated OPCAs from TAT-Cre treated cultures and allowed OPCs to migrate outwards for 10 h. Cre-recombined OPCs could be identified by their expression of EGFP, although non-recombined (tdTomato^+^) OPCs were still a significant component of the TAT-Cre treated OPCA (Fig. 4e). In sum, our work shows that the OPCA assay can be used in conjunction with conditional knockout genetics, and we next aimed to utilize this strategy to investigate the role of ILK in OPC migration.

### Loss of ILK mildly impairs OPC migration on laminin

Merosin, a blanket term for α_2_-chain containing laminin (Ln) proteins (Ln-2, Ln-4 and Ln-13; [26] influences various aspects of OL biology including growth factor-mediated survival [27, 28], morphological development [29], and CNS myelination [24]. Notably, myelinating CNS white matter tracts express the Ln α_2_-chain [30, 31], which is also upregulated after experimentally induced demyelination [32]; reviewed by [33]. In addition, Ln promotes OPC migration [34–36], rendering Ln signaling pathways appealing pharmacological targets for overcoming the inhibitory barriers impeding OPC infiltration into MS lesions.

**Fig. 5:**
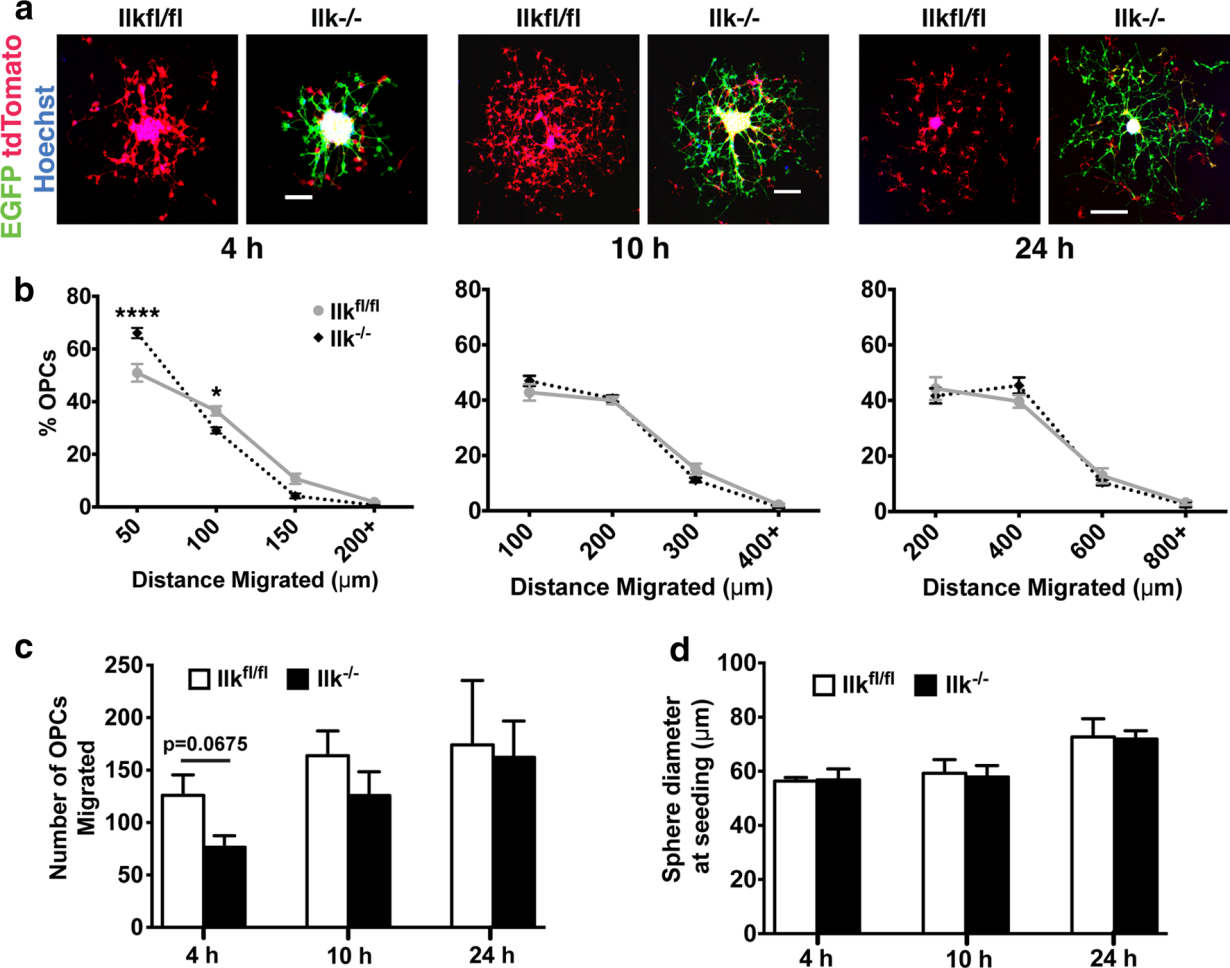
Transient impairment of OPC migration on laminin in response to ILK loss. **a** Representative confocal images of *Ilk* ^−*/*−^ and *Ilk* ^*fl/fl*^ OPCAs migrated on Ln for 4, 10 and 24 h. **b** Quantification of OPC migration using the concentric ring method (see “Methods” section). At the 4 h time point, *Ilk* ^−*/*−^ OPCs accumulated in the most proximal concentric ring (50 μm) and fewer attained the more distant 100 μm ring as compared to *Ilk* ^*fl/fl*^ control cells. Interestingly however, *Ilk* ^−*/*−^ and *Ilk* ^*fl/fl*^ OPCs migrated to the same extent at the 10 and 24 h time points. **c** No significant difference between the total number of migrated *Ilk* ^−*/*−^ and *Ilk* ^*fl/fl*^ OPCs was found at any time point. **d** There was no significant difference in initial diameter of *Ilk* ^−*/*−^ and *Ilk* ^*fl/fl*^ OPCAs. Data represent the mean ± SEM (n = 5, 5 and 4 for the 4, 10 and 24 h time points, respectively). For **b**, *p < 0.05; ****p < 0.0001 (repeated measures two-way ANOVA followed by Bonferroni post-tests). For **c** and **d**, data was tested with Student’s *t* test. *Scale bars*, 50, 100 and 200 μm for 4, 10 and 24 h time points, respectively

Ln binds the α_6_β_1_ integrin expressed by OLs [29], triggering ILK recruitment to β_1_-cytosolic tails [23], yielding Ln-α_6_β_1_-ILK complexes to which the actin cytoskeleton is stabilized. These adhesions presumably act as ECM anchor points that transmit cytoskeletal contractile forces during cellular migration. We therefore hypothesized that ILK loss would impede OPC migration on Ln due to actin-integrin disassociation. To this end, TAT-Cre treated and vehicle treated *Ilk*^*fl/fl*^*;mT/mG* OPCAs (henceforth referred to as *Ilk*^−*/*−^ and *Ilk*^*fl/fl*^ OPCAs respectively) were seeded onto Ln substrates, allowing migration to occur for 4, 10 and 24 h. Using concentric ring quantification, we found that ILK loss caused an accumulation of OPCs in the 50 μm proximal ring after 4 h compared to *Ilk*^*fl/fl*^ OPCAs. Additionally, there was a corresponding decrease in the proportion of *Ilk*^−*/*−^ OPCs that had attained the more distal 100 μm ring, ultimately indicating a reduction in net migration distance (Fig. 5a, b). Interestingly, this deficit was not evident at 10 or 24 h. There was also a near-significant trend for fewer total migrated *Ilk*^−*/*−^ OPCs at the 4 h time point (p = 0.0675; Fig. 5c), an indication of reduced capacity to initiate migration. Importantly, there was no difference between the diameters of *Ilk*^−*/*−^ and *Ilk*^*fl/fl*^ OPCAs at seeding, discounting size as a confounding variable in our observations (Fig. 5d). Rather, we attribute our findings to a role for ILK in the initiation and/or maintenance of migration on Ln.

### ILK loss severely disrupts OPC migration on fibronectin

Fibronectin (Fn) is a migration-permissive substrate for OPCs [34, 37], but evidence suggests that Fn aggregation impedes remyelination in MS [38]. This underscores the importance of elucidating the signaling pathways that govern OPC migration on Fn, as their pharmacological manipulation may influence the degree to which OPCs infiltrate MS lesions. OL-lineage cells express the α_v_β_1_, α_v_β_3_, α_v_β_5_ and α_v_β_8_ integrin receptors which recognize the Arg-Gly-Asp (RGD) integrin binding sequence intrinsic to Fn and other ECM molecules [37, 39]. As ILK is recruited to β_1_ and β_3_ integrin tails to stabilize actin at cell–matrix contacts, we hypothesized that ILK-depleted OPCs would suffer from defective migration on Fn substrates.

**Fig. 6:**
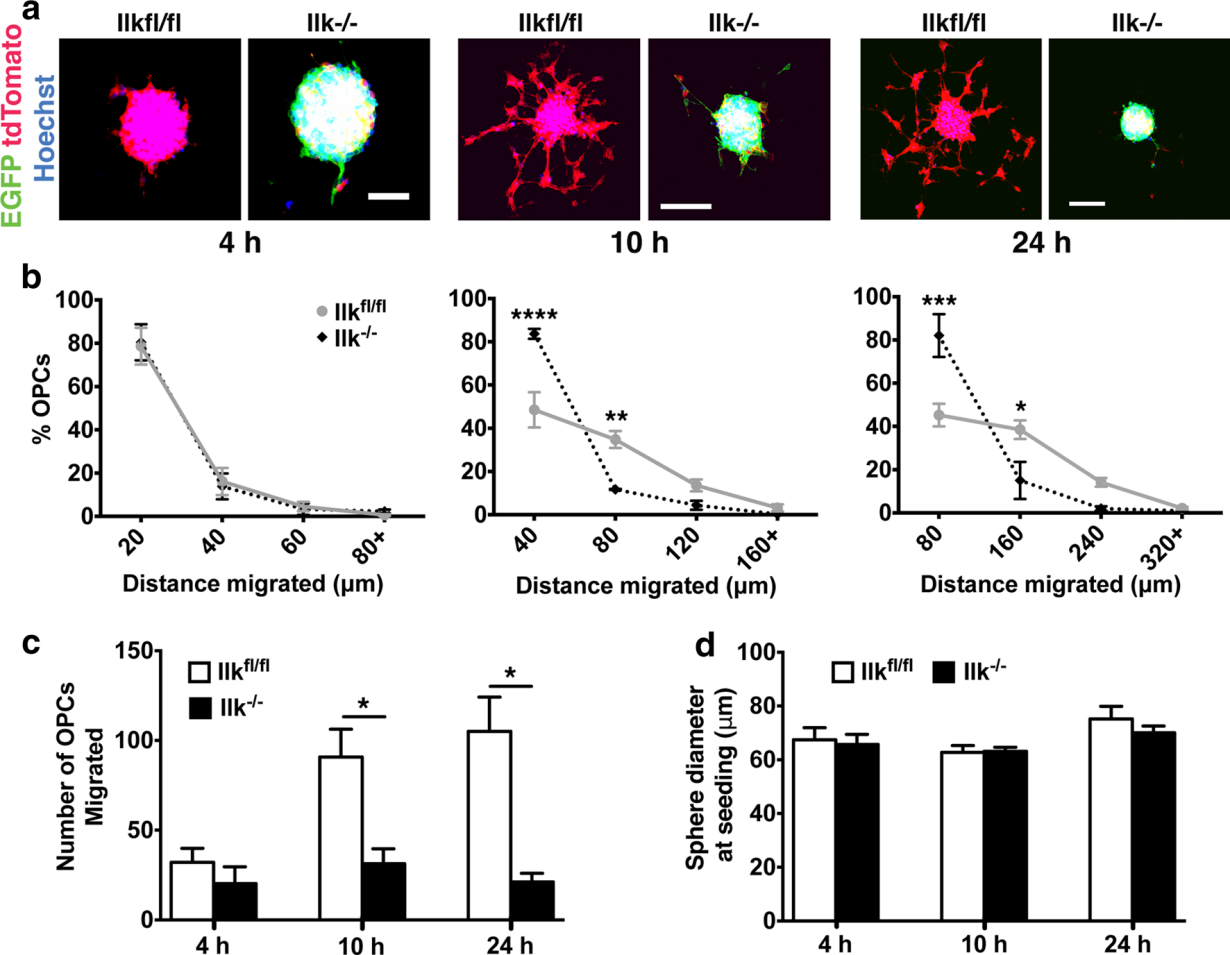
Loss of ILK perturbs OPC migration on fibronectin substrate. **a** Confocal micrographs of *Ilk* ^*fl/fl*^ and *Ilk* ^−*/*−^ OPCAs migrated on Fn for 4, 10 and 24 h. **b** Quantification revealed no difference in the distance migrated between *Ilk* ^−*/*−^ and *Ilk* ^*fl/fl*^ populations at 4 h. However, at 10 h, *Ilk* ^−*/*−^ OPCs predominantly migrated to the 40 μm proximal ring, and fewer reached the more distant 80 μm ring as compared to *Ilk* ^*fl/fl*^ cells. Similarly, at 24 h, *Ilk* ^−*/*−^ OPCs accumulated in the 80 μm proximal ring, with fewer attaining the more distal 160 μm ring. **c** There was no difference in the total number of migrated *Ilk* ^−*/*−^ and *Ilk* ^*fl/fl*^ OPCs at 4 h, while significantly fewer *Ilk* ^−*/*−^ OPCs had migrated at the 10 and 24 h time points. **d** The initial diameter of *Ilk* ^−*/*−^ and *Ilk* ^*fl/fl*^ OPCAs prior to assay commencement was not significantly different for any of the time intervals investigated. Data represent the mean ± SEM (n = 3). For **b**, *p < 0.05; **p < 0.01; ***p < 0.001; ****p < 0.0001 (repeated measures two-way ANOVA followed by Bonferroni post-tests). For **c** and **d**, *p < 0.05 (Student’s *t* test).* Scale bars*, 50 μm for 4 h, 100 μm for 10 and 24 h

*Ilk*^−*/*−^ and *Ilk*^*fl/fl*^ OPCAs were seeded onto Fn-coated coverslips, and migration was assessed after 4, 10 and 24 h. In contrast to migration on Ln, there was no substantial OPC migration of either genotype on Fn at 4 h. Not surprisingly then, we noted no migration differences between *Ilk*^−*/*−^ and *Ilk*^*fl/fl*^ OPCs at this time point (Fig. 6a, b). Rather, we observed significantly reduced net migration distance of *Ilk*^−*/*−^ OPCs at 10 and 24 h. At 10 h, *Ilk*^−*/*−^ OPCs accumulated in the most proximal concentric ring (40 μm), with correspondingly fewer cells reaching the more distal ring (80 μm), a defect that persisted at 24 h. The total number of migrated *Ilk*^−*/*−^ OPCs was also significantly reduced at 10 and 24 h (Fig. 6c), implying a role for ILK in initiating migration on Fn. We observed no significant difference in the size of *Ilk*^*fl/fl*^ and *Ilk*^−*/*−^ OPCAs at seeding time (Fig. 6e), excluding OPCA size as a factor in our analysis. Our data suggest OPC migration on Fn relies heavily on ILK, as its loss compromises both net migration distance and the initiation of migration itself.

### ILK is required for OPC migration on polylysine matrix

Poly-d-lysine (PDL) is a molecule that carries a net positive charge and interacts with anionic cell membrane domains to mediate adhesion [40]. While polylysine itself does not mediate cell adhesion via the integrins [41], it can promote the deposition of cell culture media-borne proteins [42], some of which may activate integrins to regulate migration. PDL can be thought of as a substrate that provides both a non-specific charge-based affinity for cells, and one that facilitates veritable receptor-ligand interactions.

**Fig. 7:**
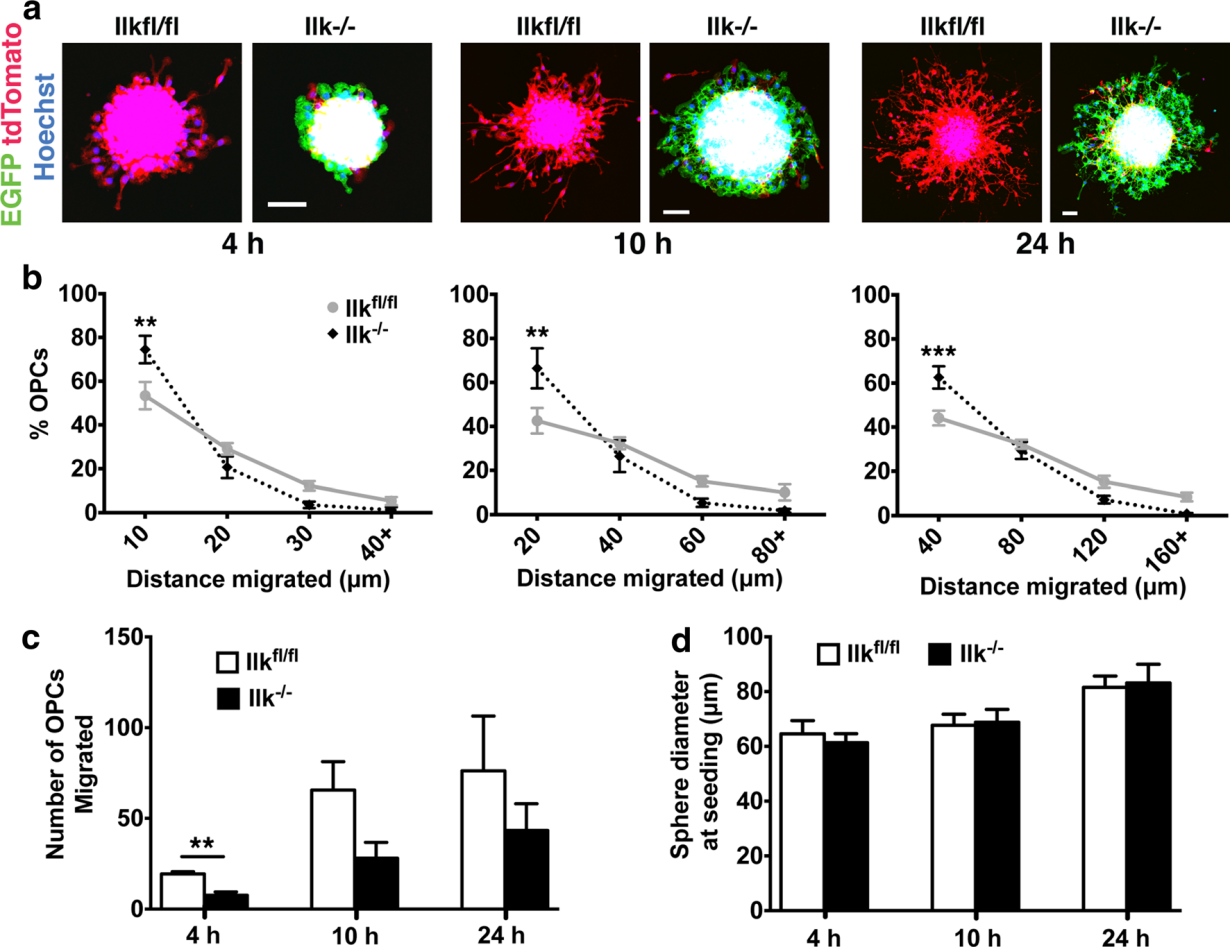
ILK mediates OPC migration on PDL. **a** Confocal images of *Ilk* ^−*/*−^ and *Ilk* ^*fl/fl*^ OPCAs migrated on PDL for 4, 10 and 24 h. **b** As compared to *Ilk* ^*fl/fl*^ OPCs, *Ilk* ^−*/*−^ OPCs were more concentrated in the 10, 20 and 40 μm proximal concentric rings at 4, 10 and 24 h respectively. **c** No difference in the total number of migrated *Ilk* ^−*/*−^ versus *Ilk* ^*fl/fl*^ OPCs was observed at 10 or 24 h, although significantly fewer *Ilk* ^−*/*−^ OPCs had migrated at the 4 h time point. **d** There was no difference in the diameters of *Ilk* ^−*/*−^ versus *Ilk* ^*fl/fl*^ OPCAs prior to commencement of the assay for any of the time points assessed. Data represent the mean ± SEM (n = 4). For **b**, **p < 0.01; ***p < 0.001 (repeated measures two-way ANOVA followed by Bonferroni post-tests). For **c** and **d**, **p < 0.01 (Student’s *t* test). *Scale bars*, 50 μm

When migrated on PDL, *Ilk*^−*/*−^ OPCs accumulated more so in the most proximal concentric rings at the 4, 10 and 24 h time points as compared to *Ilk*^*fl/fl*^ cells (Fig. 7a, b). ILK depletion also yielded fewer total migrated OPCs at 4 h, although no significant difference was observed at 10 and 24 h (Fig. 7c). This early lag in emergence from the OPCA offers a role for ILK in the initiation of OPC migration on PDL, which does not persist at later time points. Importantly, no difference was observed in the initial OPCA diameters between genotypes (Fig. 7d), excluding size as a confounding factor in our analysis. Instead, our data support an intriguing function for ILK in the initiation and maintenance of OPC migration on a substrate that likely possesses a mixture of signaling cues.

## Discussion

In this report, we demonstrate a role for ILK in OPC migration by utilizing a newly developed method tailored for mouse-derived cells. We show ILK regulates OPC migration on Ln and Fn, two ECM proteins present in demyelinated lesions [32, 38] as well as on a polylysine matrix. Impaired OPC infiltration into MS lesions is thought to partly underlie the lack of regeneration seen in chronic MS, emphasizing the need to understand the mechanics governing OPC migration, especially when pursuing remyelination therapies. Our work contributes to this increasing understanding, while also providing a new tool for investigating OPC migration that is amenable to both constitutive and conditional knockout/transgenic mouse lines.

### An assay to study mouse OPC migration in vitro

The use of mouse OPCs in basic research is becoming increasingly popular, a trend surely reflecting the accessibility of mutant lines and technological advancements in their generation. However, when compared to rat-sourced cells, the utility of mouse OPCs is limited as they are more difficult to isolate [7] and do not survive as well in culture [8]. This imposes constraints on the commonly used “agarose drop” and “transwell” migration assays, emphasizing the need for methods that account for the shortcomings of mouse OPCs. We estimate that an OPCA is composed of approximately 300-1000 cells, and a typical preparation yields roughly 50–100 OPCAs. Additionally, migration can be measured in as little as 4 h rather than several days, as is the case for the agarose drop assay. Our assay therefore accommodates the limitations of mouse OPCs by calling for few cells and requiring relatively short assay durations.

Our method bears resemblance to the “oligosphere” assay, originally described by [5]. In this method, rat OPCs are purified using a Percoll gradient, and seeded onto uncoated tissue culture vessels. Over several days, suspended OPCs form spherical aggregates reminiscent of neurospheres, while their OL identity is maintained through use of N1 supplemented B104-conditioned media. When seeded onto polyornithine substrates, OPCs emerge from oligospheres in a radial fashion as in our OPCA assay. This method has been applied to elegantly demonstrate that polysialylation of neural cell adhesion molecule (PSA-NCAM) favours OPC migration [43], and been leveraged to identify the importance of metalloproteases in OPC migration on CNS matrices [44]. To modify the assay for use with mouse cells, [11] increased the concentration of insulin and progesterone in the oligosphere culture media to enhance OPC proliferation and/or ameliorate viability [11].

One major benefit of the OPCA assay over the oligosphere method is that aggregate generation requires less researcher manipulation. To generate OPC aggregates in the OPCA assay, mixed glial cultures are simply shaken on an orbital rotator overnight in a tissue culture incubator. To generate oligospheres however, multiple 18 h differential adhesion steps need be conducted [5]. Our method also describes an efficient means to conditionally ablate/modify genes using LoxP technology, which is of high value when investigating genes whose constitutive loss leads to embryonic lethality.

Another feature of the OPCA method is its potential use in live cell imaging, facilitating the observation of OPC migration in real-time. In our hands however, we have experienced phototoxicity as a result of visualizing fluorescent proteins (e.g., EGFP, tdTomato) in live OPCs (data not shown). In addition, live imaging may not be suitable when assessing the effect of photosensitive compounds on OPC migration.

### ILK in OPC migration on various substrates

Interactions between the ECM and OL-bound integrin receptors govern numerous aspects of OL biology (reviewed by [9]. OPCs express the α_6_β_1_ integrin Ln receptor along with the α_v_β_1_ and α_v_β_3_ integrin Fn receptors [18], to which ILK binds at β_1_ and β_3_ cytoplasmic tails [19, 20]. The impaired migration of *Ilk*^−*/*−^ OPCs on Ln and Fn matrices likely results from perturbed stability of connections between the ECM and the actin cytoskeleton (Fig. 4a). Disrupting these adhesion complexes would likely translate to difficulty stabilizing a leading process, and compromise the cell’s ability to utilize focal contacts to transduce cytoskeletal contractile forces required for migration.

Interestingly, ILK loss produced only a mild deficit in OPC motility on Ln, in that migration was lagging at 4 h, but had recovered at the 10 and 24 h time points. As ILK stabilizes α_6_β_1_ integrins on Ln, our findings are in agreement with previous studies showing a role for α_6_β_1_ integrin only during the early phases of OPC migration [39], with no significant contribution in the long term [37]. These data insinuate a more significant role for α_6_β_1_ integrin in other aspects of OL biology, as it is the major β_1_-type receptor expressed by OLs, and is consistently expressed throughout OL development [18]. Accordingly, extension of OL myelin membrane [29], and CNS myelination [45, 46] are regulated by Ln-α_6_β_1_ integrin interactions, while similar functions have been identified for ILK [24, 25]. The Ln-α_6_β_1_-ILK complex may therefore be more relevant in post-migratory OL development, while only mildly contributing to Ln-directed motility. The observation that efficient migration still occurred on Ln in the absence of ILK suggests alternate signaling mechanisms predominantly drive migration on this substrate.

We offer the possibility that OPCs express additional, ILK-independent receptors that contribute to OPC migration on Ln. Existence of such receptors could explain how *Ilk*^−*/*−^ OPCs are only mildly incapacitated in their migration on Ln, and ultimately travel to the same degree as control cells. One candidate is dystroglycan, a Ln binding protein expressed by OL-lineage cells [47], with a function in OL process outgrowth [48]. Dystroglycan localizes to OL adhesion contact sites, and co-precipitates with focal adhesion kinase [48]. Recent work has also shown that nestin-Cre driven deletion of dystroglycan negatively impacts cerebellar granule neuron migration [49], offering a parallel function for dystroglycan in OPCs, as neurons and OLs share much of the same migratory mechanisms [50]. The role of dystroglycan (and/or other Ln adhesion molecules) in OPC migration remains an open question for investigation, and may provide an explanation for the migration-permissive character of Ln.

In contrast to Ln, for which α_6_β_1_ is the exclusive integrin receptor, OPCs express the Fn-sensitive α_v_β_1_, α_v_β_3_, α_v_β_5_ and α_v_β_8_ integrins [18]. With regard to our work, α_v_β_3_ and α_v_β_1_ are most relevant, as ILK exclusively binds β_1_ and β_3_ cytosolic tails [19, 20]. [39] previously demonstrated a role for α_v_β_3_ in OPC migration on Fn, while, in contrast, α_v_β_1_ was determined dispensable. A role for β_1_ integrin in OPC migration on Fn was suggested by Tiwari-Woodruff and colleagues in 2001, although in their study, migration was assessed after 4 days in vitro, a point at which OPC proliferation may confound observations. Rather, in line with [39], we favor the concept that the migratory phenotype manifested by *Ilk*^−*/*−^ OPCs on Fn is primarily a product of perturbed signaling/stability at Fn-α_v_β_3_-actin connections. Importantly, this does not discount a role for α_v_β_1_ integrin in OPC migration in a broader context, as this receptor significantly mediates OPC migration on astrocyte-produced ECM [37], a substrate surely composed of various integrin ligands. *Ilk*^−*/*−^ OPCs were not completely ablated in their ability to migrate on Fn, suggesting that receptors other than α_v_β_3_ and α_v_β_1_ may be at play. Of note, [39] previously found no role for α_v_β_5_ in OPC migration on Fn, leaving the less-characterized α_v_β_8_ integrin receptor with a possible function in this process [37].

In contrast to Ln and Fn that bind specific receptors on the cell surface, the adhesion of cells to PDL is mediated by charge interactions between anionic membrane domains and the positively charged nature of polylysine [40]. Polylysine is not thought to engage integrins, although it is capable of binding ECM proteins present in cell culture media [42]. We propose the impaired migration of *Ilk*^−*/*−^ OPCs on PDL is a result of this latter phenomenon, where ILK-devoid cells are compromised in their ability to engage PDL-immobilized ligands contributed by the media. Fetal bovine serum, which makes up 10 % of our migration media, is known to contain the ECM protein vitronectin. Similar to Fn, vitronectin also possesses the arginine-glycine-aspartic acid (RGD) motif recognized by many integrin receptors [51]. In fact, vitronectin is believed to be the major cell-attachment protein present in fetal bovine serum [52]. OLs express α_v_β_1_, α_v_β_3_, α_v_β_5_ and α_v_β_8_ integrins, which all possess affinity for vitronectin [53–56]. As ILK only binds β_1_ and β_3_ integrins, we can speculate that defective migration of *Ilk*^−*/*−^ OPCs on PDL is caused partly by the reduced stability of α_v_β_1_, α_v_β_3_ integrin adhesions responsible for stabilizing connections between vitronectin and the cytoskeleton.

### Cell signaling in OPC migration and relevance to MS therapy development

Oligodendrocyte precursor cells (OPCs) migrate extensively prior to the onset of central nervous system (CNS) myelination [57] a process governed in part by the extracellular matrix (ECM) during both normal development and remyelination of demyelinated lesions [58]. Endogenous lesion repair in MS decreases with disease progression, a consequence of reduced OPC proliferation, differentiation and/or migration [3]. Of note, OPCs accumulate at the periphery of early MS lesions [59], which is thought to be a result of lesion-derived inhibitory factors impeding OPC recruitment inwards [60–64]. Elucidating the molecular mechanisms that govern OPC migration will therefore facilitate the design of therapeutics aimed at promoting OPC infiltration into MS lesions.

We have previously shown that ILK-devoid OLs possess elevated RhoA activity, a phenomenon associated with a disorganized actin cytoskeleton [25]. This observation agrees with previous work from other cell systems [65, 66]; reviewed by [21, 22], where ILK loss leads to an upregulation in RhoA activity, while concomitantly reducing cell–matrix adhesions [67]. Conversely, reducing RhoA activity promotes reorganization of focal contacts [68], and diminishes actomyosin contractility to facilitate migration (reviewed by [69]. It is quite likely then, that an ideal level of RhoA activity exists that promotes persistent directional migration when properly localized subcellularly. In such a case, localized RhoA activity would promote cell migration by preventing the extension of numerous leading lamellipodia, ensuring focal contacts remain dynamic and by controlling the cleavage of trailing-edge adhesions. If *Ilk*^−*/*−^ OPCs do suffer from elevated RhoA activity, the dysregulation of these cellular processes could explain their compromised migratory capacity. While we did not test this hypothesis, it is an intriguing direction for future research.

The RhoA signaling axis is becoming of increasing interest with regard to MS therapeutics. Other proteins such as Netrin-1, Slit2 and NG2 mediate OPC migration through modulation of RhoA signaling [70–72]. RhoA pathway downregulation by pharmacological means enhances morphological development of OL-lineage cells [25, 73, 74], even in the presence of OL-inhibitory factors [75, 76]. Blockade of LINGO-1, an upstream regulator of RhoA (reviewed by [77], not only facilitates OL differentiation in vitro [78] but also enhances remyelination in vivo [79]. As multiple signaling pathways converge on RhoA to regulate OL biology, as well as the fact that RhoA-ROCK pathway inhibitors are currently in use for other CNS ailments [80], their efficacy in promoting remyelination in MS disease models remains an appealing direction for future investigation.

## Conclusions

Here, we present a new OPC migration assay tailored specifically for use with mouse-derived cells, which we name the OPCA assay. OPCAs are highly enriched for OPCs, and when seeded onto Ln, Fn or polylysine, rapid outward migration occurs. We further show that the OPCA method is amenable to conditional gene ablation through use of TAT-Cre recombinase to deplete ILK from OPCAs generated from *Ilk*^*fl/fl*^ mice. ILK-devoid OPCs were heavily impaired at migrating on Fn or polylysine matrices, while only mildly delayed in their migration on Ln. Inclusively, our work identifies ILK as a player in OPC migration, and provides a new tool for the study of mouse OPC motility in vitro.
